# Developing a molecular diagnostic model for heatstroke-induced coagulopathy: a proteomics and metabolomics approach

**DOI:** 10.3389/fmolb.2025.1616073

**Published:** 2025-06-20

**Authors:** Qingbo Zeng, Qingwei Lin, Longping He, Lincui Zhong, Ye Zhou, Xingping Deng, Nianqing Zhang, Qing Song, Jingchun Song

**Affiliations:** ^1^ Intensive Care Unit, The 908th Hospital of Chinese PLA Logistic Support Force, Nanchang, China; ^2^ Intensive Care Unit, Nanchang Hongdu Hospital of Traditional Chinese Medicine, Nanchang, China; ^3^ Department of Critical Care Medicine, Hainan Hospital, Chinese PLA General Hospital, Sanya, China; ^4^ Scientific Research Department, Heatstroke Treatment and Research Center of PLA, Sanya, China

**Keywords:** heatstroke, coagulopathy, machine learning, metabolomics, proteomics

## Abstract

**Background:**

Heatstroke (HS) is becoming more concerning, with coagulopathy contributing to higher mortality. The aim of this study was to analyze the metabolomic and proteomic profiles associated with heatstroke-induced coagulopathy (HSIC) and to develop a molecular diagnostic model based on proteomic and metabolomic patterns.

**Methods:**

This study included 41 HS patients from the Department of Critical Care Medicine at a comprehensive teaching hospital. Plasma proteins and metabolites from HSIC and non-heatstroke-induced coagulopathy (NHSIC) patients were compared using LC-MS/MS. Multivariate and univariate statistical analyses identified differentially expressed proteins (DEPs) and metabolites (DEMs). Functional annotation and pathway enrichment analyses were performed using the GO and KEGG databases, and machine learning models were developed using candidate proteins selected by LASSO and Boruta algorithms to diagnose HSIC. Finally, bioinformatic analysis was used to integrate the results of proteomics and metabolomics to find the potential mechanisms of HSIC.

**Results:**

A total of 41 patients participated in the study, with 11 cases in the HSIC group and 30 cases in the NHSIC group. Significant differences were observed between the groups in temperature, heart rate, white blood cell count, platelet count, liver function, coagulation markers, APACHE II score, and GCS score. Survival analysis revealed that the heatstroke group had a higher mortality risk. A total of 125 DEPs and 110 DEMs were identified, primarily enriched in energy regulation-related pathways and lipid and carbohydrate metabolism. Additionally, three optimal predictive models (AUC >0.9) were developed and validated for classifying HSIC from HS individuals based on proteomic patterns and machine learning, with the logistic regression model showing the best diagnostic performance (AUC = 0.979, sensitivity = 81.8%, specificity = 96.7%), highlighting lactate dehydrogenase A chain (LDHA), neutrophil gelatinase-associated lipocalin (NGAL), prothrombin and glucan-branching enzyme (GBE) as key predictors of HSIC.

**Conclusion:**

The study uncovered critical metabolic and protein changes linked to heatstroke, highlighting the involvement of energy regulation, lipid metabolism, and carbohydrate metabolism. Building on these findings, an optimal machine learning diagnostic model was developed to boost the accuracy of HSIC diagnosis, integrating LDHA, NGAL, prothrombin, and GBE as key biomarkers.

## 1 Introduction

HS is a severe condition marked by a rapid increase in body temperature above 40°C and central nervous system dysfunction, leading to inflammation, organ failure, and potentially death (Bouchama and Knochel, 2002; Liu et al., 2020a). It can be exertional, due to intense activity, or classic, affecting vulnerable populations like the elderly (Bouchama et al., 2022). The overall incidence of heatstroke in 2020 was 0.36 per 1,000 United States military soldiers/years (Author Anonymous, 2021), while in Japan, it ranged from 37.5 per 100,000 (95% CI, 36.8–38.2) between 2015 and 2017 to 74.4 (95% CI, 72.7–76.1) in 2018 (Ogata et al., 2021). HS is frequently accompanied by coagulation disorders and coagulopathy (Iba et al., 2022; Huisse et al., 2008). In this case, both coagulation abnormalities and HS act as contributing factors. This will worsen the patient’s condition, increase the risk of disseminated intravascular coagulation (DIC), and make treatment both challenging and less effective. Therefore, there is an urgent need to explore the risk factors and elucidating the mechanisms associated with the early diagnosis, classification of risks, and prediction of outcomes between HS and HSIC underlying their clinical development. Early identification is crucial as it facilitates prompt therapeutic intervention, potentially preventing disease progression to life-threatening complications.

Currently, we lack precise indicators and recognized diagnostic criteria to identify high-risk HSIC populations, hindering early interventions. This is mainly due to an incomplete understanding of HS’s pathogenesis and the specificity of HSIC. Researchers have analyzed the link between HS and coagulopathy, focusing on blood cells’ role in inflammation and coagulation, especially platelet dysfunction (Iba et al., 2025). Additionally, researchers found severe endothelial damage and abnormal coagulation activation during HS, and identified key coagulation and endothelial risk factors related to DIC to establish diagnostic models (Liu et al., 2025; Zeng et al., 2023). The advancement of omics technologies, such as metabolomics and proteomics, provide a novel approach for diagnosing HSIC and deepen our understanding of its occurrence and progression from an immune-metabolism perspective.

**FIGURE 1:**
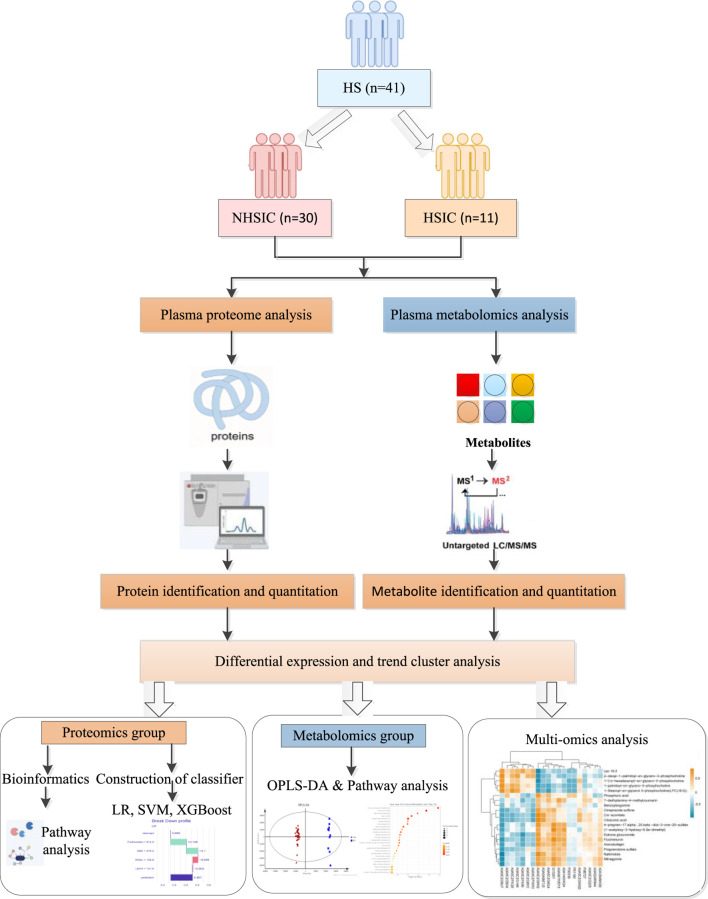
The flowchart.

In this study, we applied metabolomics and quantitative proteomics to quantitatively analyze the plasma of patients with HSIC and NHSIC (Figure 1). We identified differentially expressed metabolites (DEMs), DEPs, and key signaling pathways. Additionally, we developed diagnostic models based on proteomic patterns and machine learning. The performance of these models was evaluated using metrics such as accuracy, sensitivity, specificity, and area under the curve (AUC) to determine their potential for early detection of HSIC in clinical settings.

## 2 Materials and methods

### 2.1 Study population

This study included 41 HS patients enrolled from the Department of critical care medicine of the 908th Hospital of PLA joint logistic support force, from June 2022 to February 2024. The patients were divided into two groups: 11 cases in the HSIC group and 30 cases in the NHSIC group. The study was conducted in accordance with the ethical guidelines outlined in the Declaration of Helsinki, and informed consent was obtained from all participants or their family members. The study was approved by the ethics committee of our hospital.

Patients were diagnosed with HS or HSIC based on the Bouchama’s HS criteria (Bouchama and Knochel, 2002) and a newly proposed HSIC score (Lin et al., 2024). HS was defined by meeting the following criteria: (1) medical history of exposure to high temperature, high humidity or high-intensity exercise; (2) core temperature over 40°C; (3) CNS-related changes, including coma, convulsions, delirium, or abnormal behavior. HSIC scores were calculated according to as follows: Core temperature <40°C was scored as 0 points, ≥40°C but <42°C as 1 point, ≥42°C as 2 points; D-dimer of 2-fold increase over baseline was scored as 0 point, ≥ 2-fold but < 5-fold increase over baseline as 1 points, ≥ 5-fold increase over baseline as 2 points; prothrombin time (PT) prolongation of <2 s was scored as 0 point, ≥2 s but <4 s as 1 point, ≥4 s as 2 points. HSIC was diagnosed as a total score at least 3. Patients were excluded from our study if they were younger than 18 years old, they had a congenital coagulation disorder or chronic disease of the liver, or they were using anticoagulant drugs at the time of enrollment.

### 2.2 Sample collection process

Venous blood samples were drawn in the early morning after a minimum of 8 h of fasting and placed into a dry blood collection tube. The samples were then centrifuged at 4244 g for 10 min at 4°C. The serum obtained was separated and stored at −80°C.

### 2.3 Proteomics

In our study, we analyzed serum samples from 41 participants using DIA quantitative proteomics. The total protein from each sample was extracted, with a portion used for protein concentration determination and SDS-PAGE, while the remainder underwent trypsin digestion. After desalting, LC-MS/MS was employed to identify the peptides present. LC-MS/MS analysis was conducted on a timsTOF Pro 2 system (Bruker) coupled with Evosep One LC, employing both DDA (scan range m/z 100–1,700, ion mobility 0.75–1.35 Vs/cm^2^, 8 PASEF MS/MS cycles) for spectral library generation and 4-window DIA for quantitative profiling. Spectronaut pulsar software was used to comprehensively search the raw data and compare it against the known UniProtKB human proteome database. The false discovery rate (FDR) for both DDA and DIA data was set at 0.01. DEPs were defined with a p-value <0.05 and |log2(FC)| >1.

GO and KEGG pathway analyses were used to assess protein functions. In the GO annotation process, the protein sequences of the differentially expressed proteins were first searched locally using the NCBI BLAST + client software (ncbi-blast-2.2.28+-win32.exe) and InterProScan to identify homologous sequences. Gene ontology (GO) terms were then mapped, and the sequences were annotated using Blast2GO software, with the results visualized using R scripts. For KEGG annotation, the annotated proteins were blasted against the Kyoto Encyclopedia of Genes and Genomes (KEGG) database (http://geneontology.org/) to obtain their KEGG orthology identifications, which were subsequently mapped to relevant pathways in KEGG. A p-value less than 0.05 in the pathway enrichment test, along with a protein count greater than 5, were used as criteria for determining significance.

### 2.4 Metabolomics

Serum samples from 41 subjects were analyzed using metabolomics. Plasma samples were thawed at 4°C, and 100 µL of each sample was mixed with 400 µL of pre-cooled methanol/acetonitrile (1:1, v/v) to precipitate proteins. The mixture was centrifuged at 14,000 g and 4°C for 20 min. The supernatant was dried under vacuum centrifugation and re-dissolved in 100 µL of acetonitrile/water (1:1, v/v). After centrifugation at 14,000 g and 4°C for 15 min, the supernatant was injected for analysis. An UHPLC system (1,290 Infinity LC, Agilent Technologies) coupled to a quadrupole time-of-flight mass spectrometer (AB Sciex TripleTOF 6600) was used. Hydrophilic interaction chromatography (HILIC) was performed using a 2.1 mm × 100 mm ACQUIY UPLC BEH Amide 1.7 µm column (Waters, Ireland). In both ESI positive and negative modes, mobile phases consisted of A = 25 mM ammonium acetate and 25 mM ammonium hydroxide in water, and B = acetonitrile. The gradient started at 95% B for 0.5 min, decreased to 65% in 6.5 min, to 40% in 1 min (held for 1 min), then returned to 95% in 0.1 min with a 3 min re-equilibration. Source parameters: Gas1 = 60, Gas2 = 60, CUR = 30, temperature = 600°C, ISVF = ±5500 V. MS range: 60–1,000 Da (0.20 s/spectra); MS/MS: 25–1,000 Da (0.05 s/spectra), CE = 35 V (±15 eV), DP = ±60 V, IDA with 10 candidate ions per cycle. An UHPLC (Vanquish, Thermo) coupled to an Orbitrap (Q Exactive HF-X/Q Exactive HF) was also employed. The same HILIC column and mobile phases were used. Gradient: 98% B for 1.5 min, decreased to 2% in 10.5 min, held for 2 min, then returned to 98% in 0.1 min with a 3 min re-equilibration. MS range: 80–1,200 Da, resolution 60,000 (100 ms); MS/MS: 70–1,200 Da, resolution 30,000 (50 ms), exclude time: 4 s. Raw MS data were converted to MzXML files using ProteoWizard MSConvert, imported into XCMS for peak picking (centWave m/z = 10 ppm, peakwidth = c (10, 60)), and annotated with CAMERA. Compound identification was based on m/z and MS/MS comparison with an in-house database. The positive and negative data were merged to create a combined dataset, which was then imported into R software. After sum-normalization, the data were analyzed using the R package (ropls), performing multivariate analysis including PCA and OPLS-DA with Pareto scaling. 7-fold cross-validation and 200 response permutation testing assessed model robustness. VIP values were calculated to determine each variable’s contribution to classification. Student’s t-test was used to evaluate differences between two groups, with VIP >1 and p < 0.05 for screening significant metabolites.

### 2.5 Statistical analyses

All statistical analyses were performed using R software (version 4.1.2). Continuous variables were presented as means ± SD or medians with IQR, depending on distribution. Differences between groups were assessed using t-tests for data following a normal distribution or Mann-Whitney U tests for data following a non-normal distribution. Categorical variables were compared using chi-square or Fisher’s exact tests. A p-value <0.05 was considered significant. Feature selection was conducted with LASSO and Boruta algorithms. Predictive models were developed using LR, XGBoost, and SVM. Model performance was evaluated with metrics such as accuracy, sensitivity, specificity, AUC, kappa, and F1 score. ROC and PR curves were used to assess discriminative power, while DCA validated clinical utility. DALEX was used for model interpretability.

## 3 Results

### 3.1 Participant characteristics

A total of 41 patients with HS were enrolled in this study after screened for eligibility. All of patients with HS were divided into HSIC group (n = 11, 9 males and 2 females), and NHSIC group (n = 30, 25 males and 5 females). Inter-group comparisons revealed statistically significant differences in temperature, heart rate, white blood cells (WBC), platelets (PLT), aspartate transaminase (AST), ALT (alanine transaminase), total bilirubin (TBIL), creatinine (Cr), lactate (Lac), creatine kinase (CK), MB(myoglobin), PT, INR (international normalized ratio), APTT (activated partial thrombin time), TT (thrombin time), d-dimer, APACHE II(Acute Physiology and Chronic Health Evaluation II) score, GCS(Glasgow Coma Scale) score and MODS (multiple organ dysfunction syndrome). While age, gender, risk of coronary disease and diabetes, probability of hypertension, Hemoglobin, HCT (hematocrit), and FIB (fibrinogen) showed no statistical differences between the two groups. The comparisons between HSIC group and NHSIC group are depicted in Table 1.

**TABLE 1:** Baseline demographic.

Item	NHSIC (N = 30)	HSIC (N = 11)	p-value	q-value
Male, n (%)	25 (83)	9 (82)	>0.990	>0.990
age, yr	31 (27–36)	49 (40–58)	0.059	0.084
Temperature, °C	36.8 (36.5–37.0)	38.4 (37.7–39.2)	0.001	0.003
Heart rate, min^-1^	70 (57–80)	110 (90–124)	<0.001	<0.001
Hypertension, n (%)	0 (0)	2 (18)	0.067	0.091
Diabetes, n (%)	1 (3.3)	0 (0)	>0.990	>0.990
Coronary disease, n (%)	0 (0)	1 (9.1)	0.270	0.320
WBC, ×10^12^/L	9.0 (6.7–11.4)	14.9 (12.0–20.8)	0.009	0.015
HGB, g/L	129 (119–142)	132 (115–140)	0.660	0.710
HCT, %	39.9 (37.7–43.7)	40.5 (35.9–44.4)	0.630	0.700
PLT, × 10^9^/L	231 (178–284)	54 (30–103)	<0.001	0.001
ALT, U/L	27.1 (16.7–38.1)	110.3 (34.1–955.1)	0.001	0.003
AST, U/L	28.8 (21.7–51.4)	204.7 (65.7–476.1)	<0.001	<0.001
TBIL, μmol/L	14.3 (10.0–18.9)	22.5 (17.2–49.2)	0.003	0.005
Cr, μmol/mL	83.0 (74.8–103.2)	184.7 (104.6–299.3)	0.002	0.005
Lac, mmol/L	0.9 (0.7–1.9)	2.7 (1.800–9.8)	0.004	0.008
CK, U/L	331.7 (160.6–699.9)	1,521.0 (242.7–8,947.7)	0.009	0.014
MB, U/L	80.2 (52.1–317.3)	745.5 (580.8–852.1)	<0.001	<0.001
PT, s	13.4 (12.3–14.4)	19.1 (17.6–30.9)	<0.001	<0.001
INR	1.12 (1.05–1.20)	1.58 (1.45–2.52)	<0.001	<0.001
APTT, s	29.4 (27.9–31.4)	53.4 (37.5–120.0)	<0.001	<0.001
TT, s	16.4 (15.5–17.6)	18.9 (16.7–27.4)	0.005	0.010
FIB, g/L	2.13 (1.85–2.49)	1.93 (1.21–2.66)	0.490	0.560
D-dimer, μg/L	0.24 (0.19–0.870)	14.80 (3.75–29.97)	<0.001	<0.001
APACHE II score	11 (7–15)	28 (21–39)	<0.001	<0.001
GCS	13 (11–15)	4 (3–8)	<0.001	<0.001
MODS, n (%)	4 (13)	8 (73)	<0.001	0.002
Mortality, n (%)	1 (3.3%)	3 (27%)	0.052	0.077

Values are n (%), mean ± standard deviation or median (interquartile range), unless otherwise noted.

Abbreviations: WBC, white blood cells; HGB, hemoglobin; HCT, hematocrit; ALT, alanine transaminase; AST, aspartate transaminase; Cr, creatinine; TBIL, total bilirubin; Lac, lactate; CK, creatine kinase; MB, myoglobin; PLT, platelets; PT, prothrombin time; INR, international normalized ratio; APTT, activated partial thrombin time; TT, thrombin time; FIB, fibrinogen; APACHE II, score, Acute Physiology and Chronic Health Evaluation II; GCS, glasgow coma scale; MODS, multiple organ dysfunction syndrome.

### 3.2 APACHE II score and survival analysis in the study groups

**FIGURE 2:**
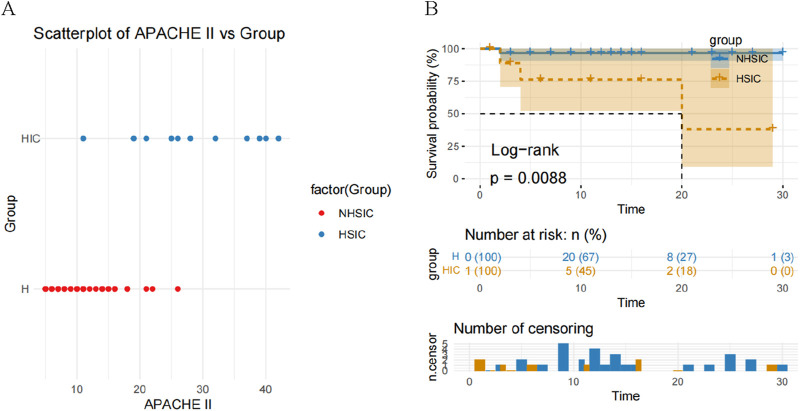
**(A)** A scatterplot of APACHE II score comparison in the NHSIC vs. HSIC group; **(B)** Kaplan–Meier curves for adverse outcome according to the study groups.

Compared with NHSIC group, the APACHE II score in HSIC group is significantly increased (Table 1; Figure 2A). The cumulative risk curve of adverse outcome according to the study groups is shown in Figure 2B. The result demonstrated that individuals in NHSIC group were at the higher risk of death compared with HSIC group.

### 3.3 Proteins identification and differential proteins analysis

**FIGURE 3:**
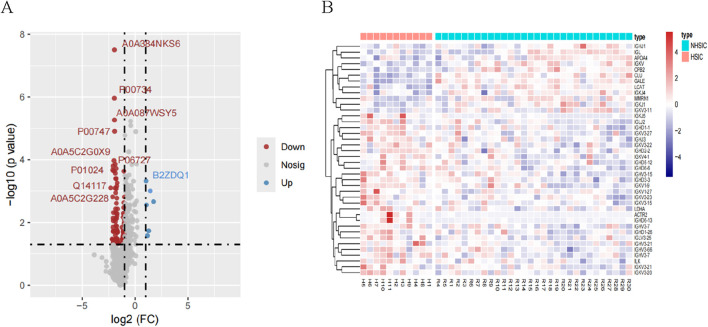
**(A)** A total of 125 differentially expressed proteins were screened in the NHSIC vs. HSIC group (6 upregulated and 119 downregulated). **(B)** Cluster analysis of DEPs in the group of NHSIC vs. HSIC.

We identified a total of 1907 proteins. For differential expression analysis, p value <0.05 were considered statistically significant and |log2(FC)| >1 was considered up- or downregulated, respectively. A total of 125 DEPs were screened in the NHSIC group vs. HSIC group. A comparison of NHSIC and HSIC patients found that there were 6 and 119 upregulated and downregulated proteins, respectively (Figure 3A). Hierarchical clustering algorithm was used to perform cluster analysis on the DEPs of NHSIC and HSIC groups, and the data was displayed in the form of heat map (Figure 3B).

### 3.4 Functional analysis of differentially expressed proteins

**FIGURE 4:**
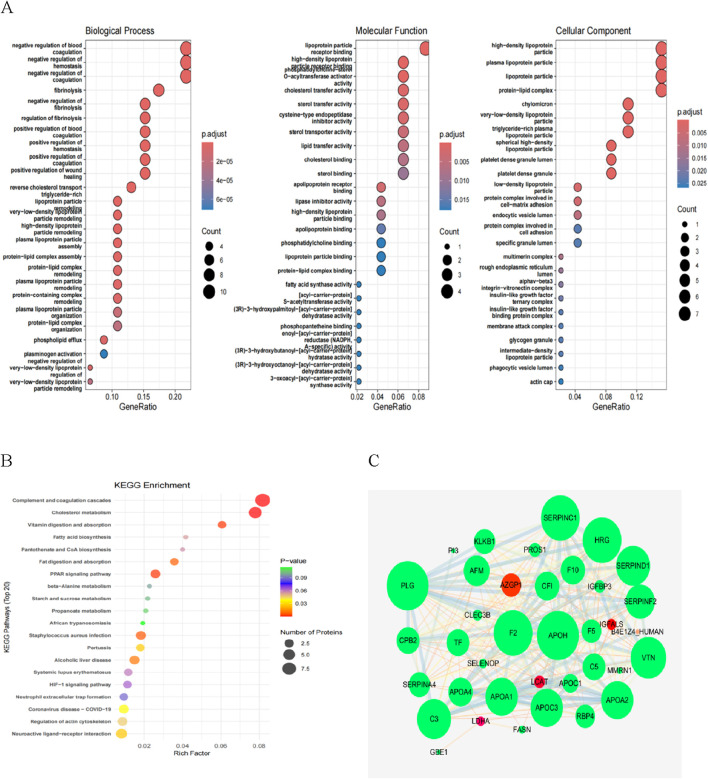
Functional enrichment analysis of NHSIC vs. HSIC differentially expressed protein. **(A)** The enriched GO functional classification, which is divided into three major categories: Biological Process (BP), Molecular Function (MF), and Cellular Component (CC). The color of the bar graph indicates the significance of the enriched GO functional classification, which is based on Fisher’s accuracy; Fisher’s Exact Test calculated the P value. The color gradient represents the size of the P value, from red to blue; the closer to blue, the smaller the P value, and the higher the significance level of the enrichment of the corresponding GO function category. **(B)** The DEPs mainly concentrated in complement and coagulation cascades, cholesterol metabolism, and neuroactive ligand−receptor interaction. **(C)** DEP interaction networks in group of NHSIC vs. HSIC.

We performed GO functional annotation on the differentially expressed proteins (DEPs) (Figure 4A). In the NHSIC vs. HSIC group, the difference in protein expression was greatest in Biological Process (BP), Molecular Function (MF), and Cellular Component (CC). Concentrated results were in negative regulation of coagulation, lipoprotein particle receptor binding, and lipoprotein particle. We used KEGG to analyze the signaling pathways of 125 DEPs in the NHSIC vs. HSIC group that were mainly concentrated in complement and coagulation cascades, cholesterol metabolism, and neuroactive ligand−receptor interaction (Figure 4B). From the 125 DEPs in the NHSIC vs. HSIC group, 36 proteins were involved in protein interactions. Furthermore, PPIs analysis showed that APOH and PLG had the highest degree scores in the network (Figure 4C), suggesting that they play a role in the pathological process of HS.

### 3.5 Identification and further analysis of candidate DEPs involved in HSIC

**FIGURE 5:**
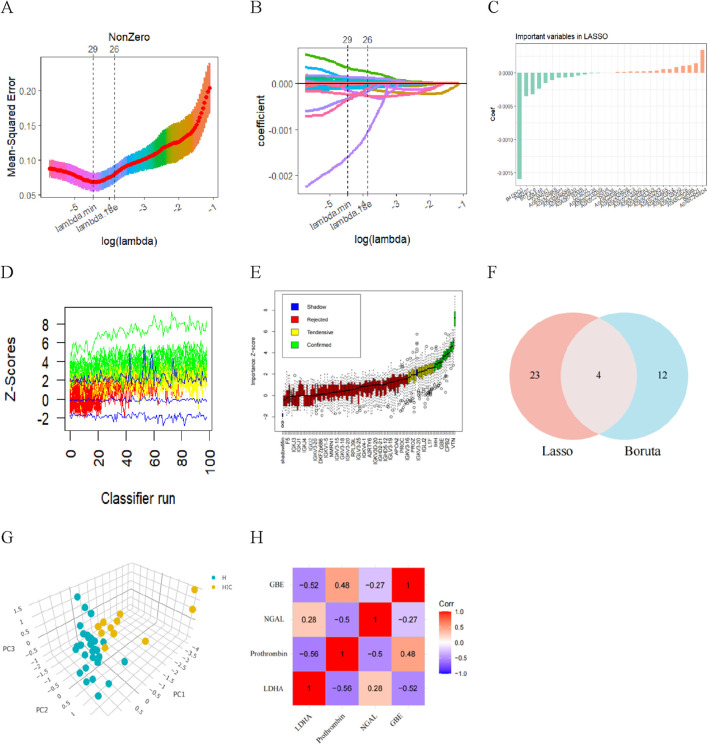
Diagnostic indicators for HSIC screening. **(A)** Fine-tuning the least absolute shrinkage and selection operator (LASSO) model’s feature selection. The ordinate represents the value of the coefficient, the lower abscissa represents log (l), and the upper abscissa represents the current number of non-zero coefficients in the model. **(B)** LASSO coefficient profiles. **(C)** The important indicators in Lasso. **(D)** History of decisions of rejecting or accepting features by Random Forest in 100 Boruta function runs. **(E)** Boxplot of all features from random forest analysis, with green indicating important variables, while red, blue, and yellow represent rejected variables. **(F)** Venn diagram showing overlapping markers. **(G)** Principal component analysis shows that the four proteins aforementioned can clearly distinguished NHSIC and HSIC. **(H)** The correlation among LDHA, Prothrombin, NGAL, GBE.

LASSO regression introduces a penalty function to continuously compress the coefficients, streamline the model, and avoid multicollinearity and overfitting, thereby achieving the effect of variable selection. When a standard error of the minimum distance λ was 0.012, 27 feature coefficients were nonzero (Figures 5A–C). The process and results of feature selection using Random Forest are shown in Figures 5D,E, which identified 16 important variables predisposing to HSIC in heatstroke patients. The two methods identified overlapping proteins, two upregulated (LDHA and NGAL), two downregulated (Prothrombin and GBE) (Figure 5F). Then, these four most vital variables were selected for further analysis. As the principal component analysis result in Figure 5G, the four DEPs aforementioned can clearly distinguished NHSIC and HSIC, which indicated that they may play key roles in the diagnosis of HSIC. The correlations of the proteins were also analyzed as shown in Figure 5H. The absence of significant correlations among these proteins indicates they do not exhibit functional similarities.

### 3.6 Construction and assessment of XGBoost, LR and SVM model for HSIC diagnosis

**FIGURE 6:**
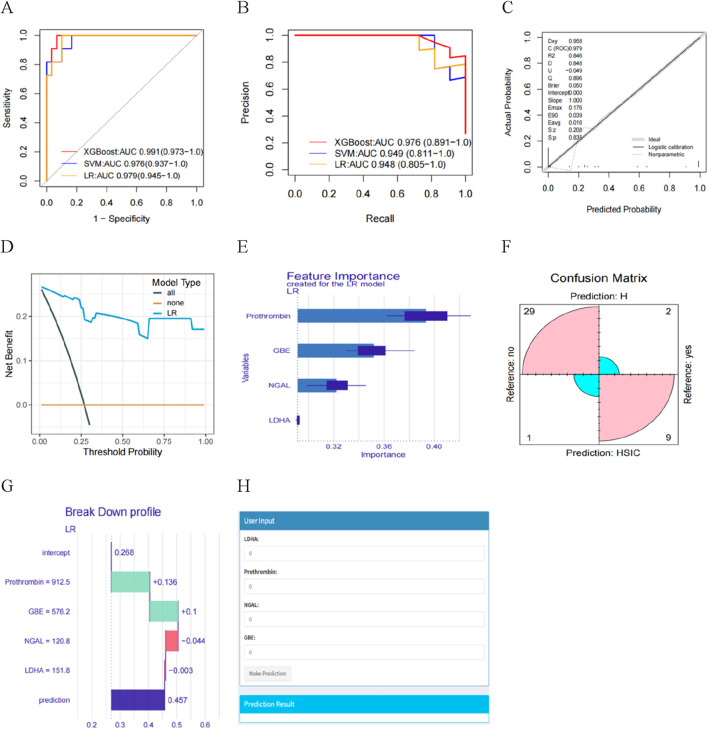
**(A)** The AUC of the three models. **(B)** Learning curve. **(C)** Calibration curves for the LR model for predicting HSIC probability. **(D)** Decision curve analysis evaluating the clinical benefit of the predictive model. **(E)** Feature importance derived from LR model. **(F)** Confusion matrix showing the classification accuracy. **(G)** Explaining of patient prediction results. **(H)** User-friendly interface of the LR model facilitating HSIC probability prediction.

XGBoost, LR and SVM algorithms were used to construct models based on selected proteins. We used AUC, accuracy, no information rate, balanced accuracy, kappa, sensitivity, specifcity, precision, and F1 scores to comprehensively evaluate the model’s performance. XGBoost had the largest AUC (0.991) and precision (1.0), followed by LR (AUC: 0.979; precision: 0.90) and SVM (AUC: 0.976; precision: 0.750) (Table 2). Figure 6A described the ROC curves for the three models. The accuracy, kappa, sensitivity, Balanced accuracy and F1 scores of LR were higher than those of XGBoost and SVM, as shown in Table 2. Therefore, The LR had better clinical utility compared to XGBoost and SVM.

**TABLE 2:** Performance of three machine learning models for predicting HSIC in critically ill patients.

Models	AUC	Accuracy	No information rate	Balanced accuracy	Kappa	Precision	F1 score	Sensitivity	Specificity
XGBoost	0.991	0.877	0.731	0.773	0.637	1.000	0.706	0.545	1.000
SVM	0.976	0.878	0.730	0.859	0.698	0.750	0.783	0.818	0.900
LR	0.979	0.927	0.732	0.892	0.808	0.900	0.857	0.818	0.967

AUC, area under curve; LR, logistic regression; XGBoost, eXtreme gradient boosting; SVM, support vector machine.

The ROC curves and PR curves for the three models indicated that the LR was better than XGBoost and SVM in discrimination (Figures 6A,B). The calibration curve fit was good (Figure 6C), indicating that the model had good diagnostic performance. Furthermore, we found the net benefits (NB) of LR model was higher than 0, with the greater NB in DCA clinical evaluation (Figure 6D), indicating the importance of the LR model for HSIC diagnosis. The DALEX package was used for logistic regression analysis to further demonstrate the importance of the four proteins in the model and the descending order of importance of these features in the model was LDHA, NGAL, Prothrombin and GBE (Figure 6E). Based on the confusion matrix, the assay had a sensitivity, specificity, PPV, and NPV of 81.8%, 96.7%, 90.0%, and 93.6% respectively (Figure 6F).

### 3.7 Model explainability and visualization

We present one instances in which interpretability analyses of the model predictions. The ML model predicted a 45.7% risk of HSIC based on four critical predictors. We found prothrombin and GBE being the two contributors to the increased risk of HSIC, whereas LDHA and NGAL reduced the model’s diagnosis of HSIC (Figure 6G). We developed the interface to facilitate the use of the model to explore the relative contribution of HSIC probability factors in ICU patients. In the diagnosis view, the system invokes a diagnosis model, and the LR model diagnoses the patient’s HSIC. The analysis results are visualized in a graphic view, which indicates the HIC probability of the patient input important features values (Figure 6H).

**FIGURE 7:**
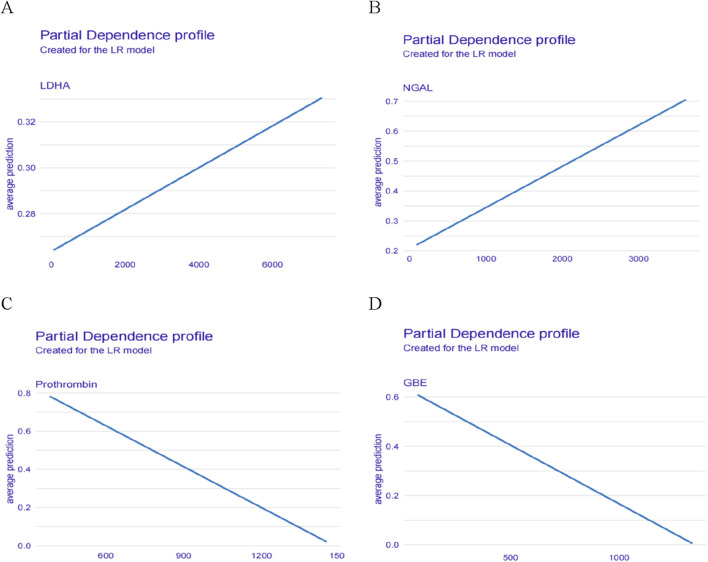
Relationship between **(A)** LDHA, **(B)** NGAL, **(C)** Prothrombin, **(D)** GBE and predicted HSIC.

In addition, we also described the effect (positive or negative) of four proteins on the model. Figure 7 showed the relationship between LDHA, Prothrombin, NGAL, GBE and predicted HSIC. Higher LDHA and NGAL were associated with an increased risk of HSIC. Lower Prothrombin and GBE were associated with an increased risk of HSIC (Figure 7).

### 3.8 Metabolomic analysis

**FIGURE 8:**
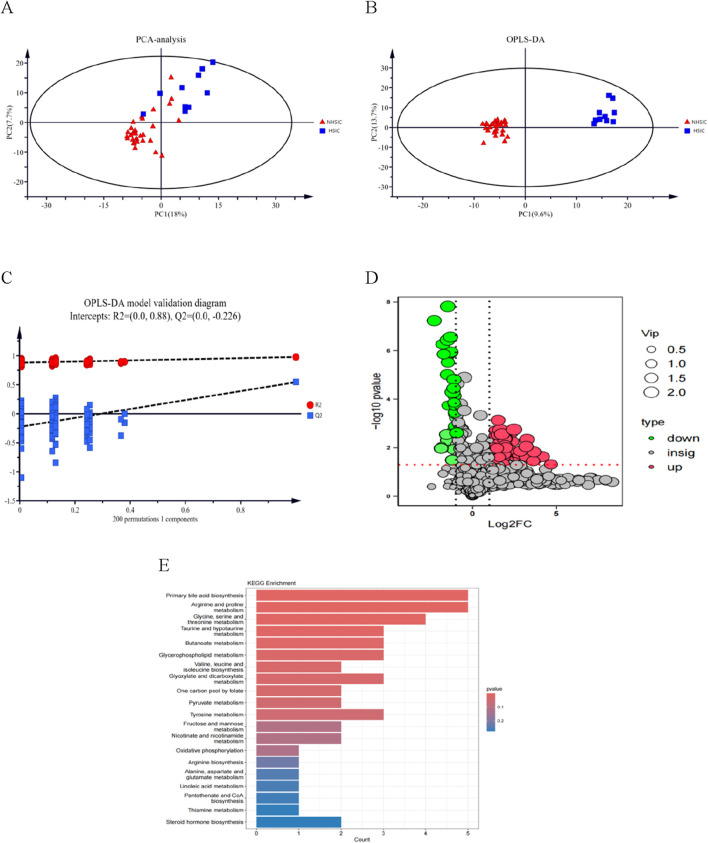
**(A)** Score chart of PCA analysis. **(B)** Score chart of OPLS-DA analysis. **(C)** PLS-DA model validation diagram. **(D)** A volcano plot of the differential metabolites. **(E)** A bubble diagram of top-25 metabolic pathways.

Figure 8A displays the results of principal component analysis (PCA), which clearly separates the samples into distinct groups. Figure 8B presents the OPLS-DA scores, highlighting a significant difference between the two groups. The robustness of the OPLS-DA model was validated through permutation analysis, yielding an R^2^Y of 0.979 and a Q^2^ value of 0.545. In Figure 8C, the negative Q^2^ value confirms the absence of over-fitting, further validating the model’s reliability and effectiveness. Differential expression analysis, based on a Benjamini–Hochberg adjusted filter of <0.05 and a log2 fold change (FC) > 1.0, revealed 110 significantly DEMs, including 30 upregulated and 80 downregulated metabolites. These 110 DEMs are shown in the volcano plot in Figure 8D. Finally, Figure 8E illustrates the top 25 enriched metabolic pathways for these DEMs, as identified using the KEGG database.

### 3.9 Bioinformatic analysis-integrated analysis of proteomics and metabolomics

**FIGURE 9:**
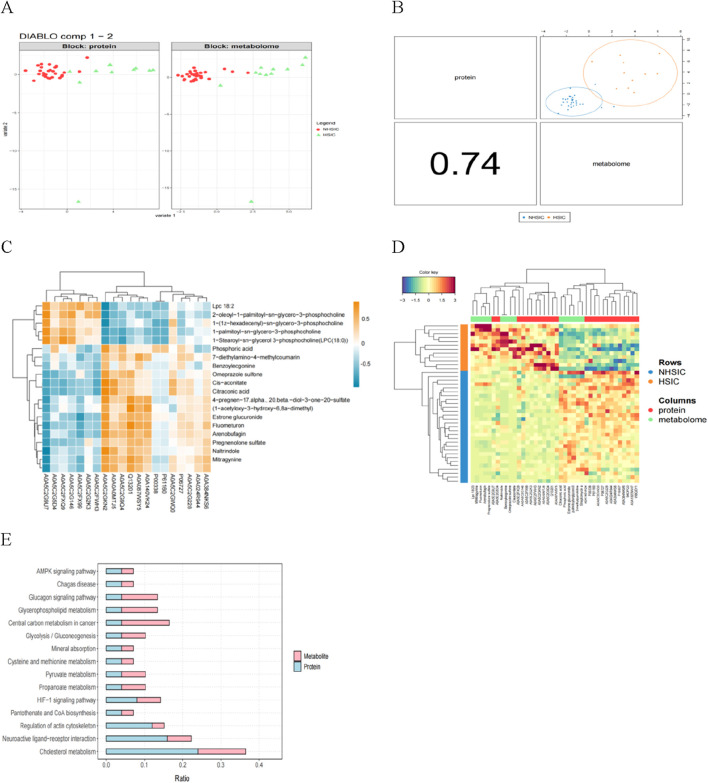
**(A)** The proteomics (left) and metabolomics (right) features can clearly distinguish the samples. **(B)** The multi-omics correlation plot shows the Pearson correlation between proteomics and metabolomics, supporting their integration and the presentation of a joint signature. **(C)** Correlation analysis of the differential proteins and metabolites. **(D)** The multi-omics clustering heatmap is structured such that samples are displayed in rows and molecular features (e.g., proteins, metabolites) are arranged in columns. **(E)** KEGG pathway annotation of differential proteins and metabolites.

Through DIABLO algorithm, we identified distinct clustering patterns differentiating NHSIC and HSIC samples (Figure 9A). Subsequent integrative modeling of multi-omics features revealed strong cross-correlations between proteomic and metabolomic profiles, highlighting their coordinated biological regulation (Figure 9B). To identify significant protein-metabolite interactions, we extracted the top 20 differentially expressed proteins (DEPs) and metabolites (DEMs) ranked by p-values and performed Pearson correlation analysis to evaluate their associations, followed by the generation of a correlation heatmap (Figure 9D). Multi-omics clustering analysis revealed strong inter-dataset associations between features derived from the proteomic and metabolomic profiles (Figure 9D). Additionally, the differentially expressed proteins and metabolites were mapped to the KEGG database to assess the potential relationship. It was found that the AMPK signaling pathway, cholesterol metabolism, glycolysis/gluconeogenesis, neuroactive ligand-receptor interaction, propanoate metabolism, glucagon signaling pathway, glycerophospholipid metabolism, and pantothenate and CoA biosynthesis were significantly enriched (Figure 9E).

## 4 Discussion

This study explored the clinical characteristics, biomarkers, and the performance of predictive models for HSIC. It revealed significant differences in clinical markers such as APACHE II scores, liver function, and coagulation markers between the NHSIC and HSIC groups. There were 125 DEPs and 110 DEMs identified in the comparison between the NHSIC and HSIC groups and Significant protein and metabolite alterations were found primarily in energy regulation and lipid metabolism pathways. Specific proteins, such as LDHA, NGAL, GBE, and prothrombin, were identified as potential biomarkers for HSIC diagnosis. Additionally, Machine learning models, including XGBoost, LR, and SVM, were developed for HSIC diagnosis, with the LR model showing the best diagnostic performance.

The clinical markers, including temperature, heart rate, AST, ALT, PT, and INR, observed in both the NHSIC and HSIC groups, provide valuable insights into the pathophysiology of HS and its complications. These variations align with previous studies showing that heatstroke causes organ dysfunction and coagulation abnormalities (He et al., 2022; Zhong et al., 2021). The higher APACHE II scores in the HSIC group further highlight the connection between the severity of heatstroke and poor prognosis, as high APACHE II scores are known to predict adverse outcomes (Shimazaki et al., 2020). The identification of 125 DEPs, reflecting changes in coagulation and lipoprotein metabolism, supports earlier studies (Li et al., 2023; Caputa et al., 2000). The functional analysis of these proteins emphasized the role of key signaling pathways, including complement and coagulation cascades. Heatstroke, a septic-like condition, involves these pathways, which are crucial in both sepsis and HSIC (Jiang et al., 2024; Liu et al., 2020b). Subsequently, a metabolomic analysis was performed and 103 DEMs were identified. Consistent with the proteomics results, we observed significant regulation of energy metabolism-related biological pathways. Multiomics analysis of metabolomics and proteomics datasets based on the same biological samples was applied in this study. It was found that HS is closely associated with energy regulation-related pathways as well as lipid and carbohydrate metabolism. Machine learning models showed better performance in diagnose HSIC, further supporting previous research in this area (Umemura et al., 2024; Zeng et al., 2023).

The main academic contribution of this study lies in providing new insights into the diagnosis of HSIC through the analysis of clinical data and proteomics data, combined with machine learning methods. First, our study identified and analyzed 125 DEPs and 100DEMs, revealing significant differences in protein and metabolite expression between NHSIC and HSIC, particularly in pathways related to lipid and carbohydrate metabolism. Secondly, through LASSO regression and random forest analysis of these differentially expressed proteins, four key proteins -LDHA, NGAL, GBE, and Prothrombin -were identified. LASSO is a widely used regression method for high-dimensional data analysis that automatically selects variables through L1 regularization. It is particularly suitable when the number of features far exceeds the sample size, improving model predictive performance and enhancing interpretability. Boruta, a random forest-based feature selection algorithm, systematically evaluates feature importance to ensure optimal predictivity and interpretability. Combining LASSO with Boruta results in a more stable model with stronger interpretability. LDHA activation under hypoxia/inflammation drives lactate accumulation, which signals through endothelial GPR81 to increase vascular permeability and amplify systemic inflammation (Huau et al., 2024; Chen et al., 2023; Khatib-Massalha et al., 2020). Additionally, the metabolic product of LDHA, lactate, can affect endothelial cell permeability and coagulation function (Yang et al., 2022). NGAL, an acute-phase protein, is upregulated in inflammatory responses (Chakraborty et al., 2012). NGAL is not only involved in the activation of neutrophils but also promotes the vicious cycle of thrombus formation and inflammatory responses through its interaction with the coagulation system (Xue et al., 2025). Heatstroke can also cause renal damage, so its levels are correlated with the severity of heatstroke (Chen et al., 2024). GBE is closely related to lipid metabolism, and abnormalities in lipid metabolism during heatstroke may lead to changes in its levels. Prothrombin is a pivotal protein within the coagulation system. Its accelerated activation under inflammatory conditions can precipitate coagulation dysfunction, elevate the risk of thrombosis, and further intensify inflammatory responses (Teodoro et al., 2023). Moreover, growing evidence suggests that heparanase may contribute to heatstroke-induced coagulopathy by causing endothelial damage and glycocalyx degradation, which enhances tissue factor (TF) exposure, promotes prothrombin activation, and leads to excessive coagulation activation (Capozzi et al., 2021). Since heatstroke is frequently associated with coagulation abnormalities, changes in prothrombin are associated with HSIC (Degen and Sun, 1998; Wright et al., 1946). Based on these four important proteins, several machine learning models (XGBoost, LR, and SVM) were developed for the diagnosis of HIC. Comprehensive performance evaluations showed that the logistic regression (LR) model demonstrated the best diagnostic ability in clinical applications, with high sensitivity, specificity, and clinical utility, enabling early diagnosis and risk assessment of HSIC. However, this study has limitations that should be addressed in future research. The sample size of 41 patients is relatively small, which may limit the generalizability of the findings. Additionally, the study focused on a limited set of clinical and proteomic markers; further studies could explore a broader range of biomarkers and clinical variables. Longitudinal data is needed to validate the prognostic value of the identified biomarkers and the performance of the machine learning models in predicting long-term outcomes. Lastly, the interpretability of machine learning models, while explored in this study, could benefit from further refinement to enhance clinical utility, ensuring that these models are both accurate and explainable for healthcare providers.

In summary, this study provides valuable insights into the clinical and molecular mechanisms of HSIC and demonstrates the utility of machine learning models in improving the diagnosis and diagnosis of HSIC. Future research should aim to validate these findings in larger cohorts and explore additional biomarkers that could further enhance the understanding and management of HSIC.

## Data Availability

The data presented in the study are deposited in the ProteomeXchange repository, accession number PXD064588.
